# Genetic variants of interferon-response factor 5 are associated with the incidence of chronic kidney disease: the D.E.S.I.R. study

**DOI:** 10.1038/s41435-023-00229-4

**Published:** 2023-11-17

**Authors:** Frédéric Fumeron, Gilberto Velho, Fawaz Alzaid, Ray El Boustany, Claire Vandiedonck, Amélie Bonnefond, Philippe Froguel, Louis Potier, Michel Marre, Beverley Balkau, Ronan Roussel, Nicolas Venteclef

**Affiliations:** 1 Université Paris Cité, INSERM UMR-S1151, CNRS UMR-S8253, Institut Necker-Enfants Malades, Paris, France; 2 Dasman Diabetes Institute, Kuwait City, Kuwait; 3 Inserm U1283, CNRS UMR 8199, European Genomic Institute for Diabetes (EGID), Institut Pasteur de Lille, Lille, France; 4 University of Lille, Lille University Hospital, Lille, France; 5 Department of Metabolism, Digestion and Reproduction, Imperial College London, London, UK; 6 Department of Diabetology, Endocrinology and Nutrition, Assistance Publique-Hôpitaux de Paris, Bichat Hospital, DHU FIRE, Paris, France; 7 Clinique Ambroise Paré, Neuilly-sur-Seine, France; 8 Centre for Research in Epidemiology and Population Health (CESP), INSERM, UMR-S 1018, University Paris-Sud, University Versailles Saint-Quentin, Villejuif, France

**Keywords:** Genetics, Genetic association study

## Abstract

Inflammation has been associated with renal diseases. The Interferon Regulatory Factor (IRF)-5 is a key transcription factor in the pro-inflammatory polarization of M1-like macrophages. GWAS have reported that the *IRF5* locus is associated with autoimmune diseases and with the estimated glomerular filtration rate (eGFR). We study whether allelic variations in *IRF5* are associated with the incidence of chronic kidney disease (CKD) in a general population. We genotyped eleven *IRF5* SNPs in the French D.E.S.I.R. cohort from the general population (n = 4820). Associations of SNPs with baseline renal parameters were assessed. Data were analyzed for three endpoints during a 9-year follow-up, incidence of:at least stage 3 CKD, the KDIGO criterion “certain drop in eGFR”, and incidence of micro/macro albuminuria. In the cross-sectional analysis, rs10954213 and rs10954214 were associated with eGFR and rs1874328 with urinary albumin/creatinine ratio (ACR). Rs3807306, rs11761199, rs78658945, rs1874328, rs10954213 and rs11770589 were associated with the incidence of stage 3 CKD in multi-adjusted models. Rs4731532, rs3807306, and rs11761199 were associated with the incidence of CKD defined by the KDIGO. Rs4731532, rs3807306, rs11761199 and rs79288514 were associated with the incidence of micro/macro albuminuria. Our results support the hypothesis of the importance of IRF5 mediated macrophage polarization in the etiology of CKD.

## Introduction

Chronic inflammation forms part of virtually every human disease, including renal diseases [1, 2]. Macrophages are key players in the inflammatory process: upon M1-like polarization, they secrete powerful proinflammatory cytokines [3]. These molecules exert their effects on neighboring parenchymal cells and recruit monocytes from circulation that can amplify local inflammation [3]. Macrophages are the main effector cells of kidney inflammation, their M1-like polarization is a characteristic feature of chronic inflammation: recruited monocytes infiltrate the kidney and differentiate to increase macrophage numbers in the tissue [1, 4, 5]. This has been reported in human chronic kidney disease (CKD). In addition, in experimental progressive CKD, M1-like macrophages are present from the early phases of inflammation [4], and the magnitude of macrophage infiltration correlates with the severity of kidney injury [6–8]. Despite these reports suggesting an effector role for macrophage infiltration and M1-like polarization in renal disease, the events initiating M1-like polarization in human CKD require further elucidation.

Under healthy physiological conditions, the kidney macrophage compartment includes a population of phenotypically distinct resident macrophages as well as a minority population of macrophages differentiated from circulating monocytes [9–11]. The latter compartment undergoes rapid expansion upon tissue injury, and this occurs in a number of disease contexts, including kidney disease [11]. As reviewed in [1], most forms of acute renal inflammation feature macrophage infiltration with a predominant M1-like phenotype.

M1-like macrophage polarization is transcriptionally controlled by the interferon regulatory factor (IRF)-5 [12], a transcription factor that pioneers the type-1 interferon (IFN-I) response, orchestrating both acute and chronic inflammation [13]. Our own studies have shown that IRF5 is metabolically responsive, and its dysregulated activity plays a role in adipose tissue and liver inflammation upon insulin resistance [14–16].

In genome-wide association studies (GWAS), variants at the *IRF5* locus have been associated with autoimmune diseases (systemic lupus erythematous, rheumatoid arthritis, multiple sclerosis) [17–19]. Wuttke et al. [20] demonstrated in a large GWAS meta-analysis, of more than 1 million individuals, that an *IRF5* polymorphism was associated with the estimated glomerular filtration rate (eGFR). Given this result, the known function of IRF5 in macrophage polarization and the role of macrophages in renal disease, we sought to investigate whether *IRF5* variants are associated with kidney disease.

To test the hypothesis that allelic variants in *IRF5* are associated with the incidence of kidney function related outcomes in the general population, we explored the impact of *IRF5* genetic variations in a longitudinal study, the D.E.S.I.R. (Data from an Epidemiological Study on the Insulin-Resistance syndrome) cohort.

## Methods

### Population

The D.E.S.I.R. study is a prospective study of 5212 unrelated participants at inclusion (2576 men and 2636 women, aged 30 to 65 years), recruited from volunteers who were offered periodic health examinations free of charge by the French Social Security system in 10 health examination centers from the western part of France. They were clinically and biologically evaluated at 3-yearly visits and the final examination was 9 years after inclusion. A detailed description of all clinical and laboratory measurements has been reported [21].

To avoid population stratification problems, only individuals born in mainland France were kept for genetic analyses (n = 4820). The D.E.S.I.R. study was approved by the ethics committee of the Kremlin Bicêtre Hospital, and all participants signed an informed consent according to European legislation.

The estimated glomerular filtration rate (eGFR) was calculated using serum creatinine concentrations and the CKD-EPI (Chronic Kidney Disease Epidemiology collaboration) equation [22, 23]. Urinary albumin and creatinine were assayed in about 3/4 of the sample (n = 3698), to calculate the urinary albumin/creatinine ratio (ACR).

Participants were followed for eGFR decline and for new-onset chronic kidney disease (CKD) during a median (IQR) duration of 9.0 (0.6) years. We considered three criteria for kidney function decline and progression during follow-up:

1. the incidence of at least stage 3 CKD — defined as an eGFR below 60 ml/ min/1.73 m² — in at least one of the follow-up visits
2. a “Certain Drop in eGFR” criterion proposed by the KDIGO group [24]; six eGFR categories were defined as ≥90, ([90, 60]), ([60, 45]), ([45, 30]), ([30, 15]), and eGFR < 15 ml/min/1.73 m². A “Certain Drop in eGFR” was defined by KDIGO as a drop in eGFR category accompanied by a 25% or greater drop in eGFR from baseline
3. the incidence of micro/macro albuminuria — defined as ACR ≥ 30 mg/g — in at least one of the follow-up visits.

**Fig. 1:**
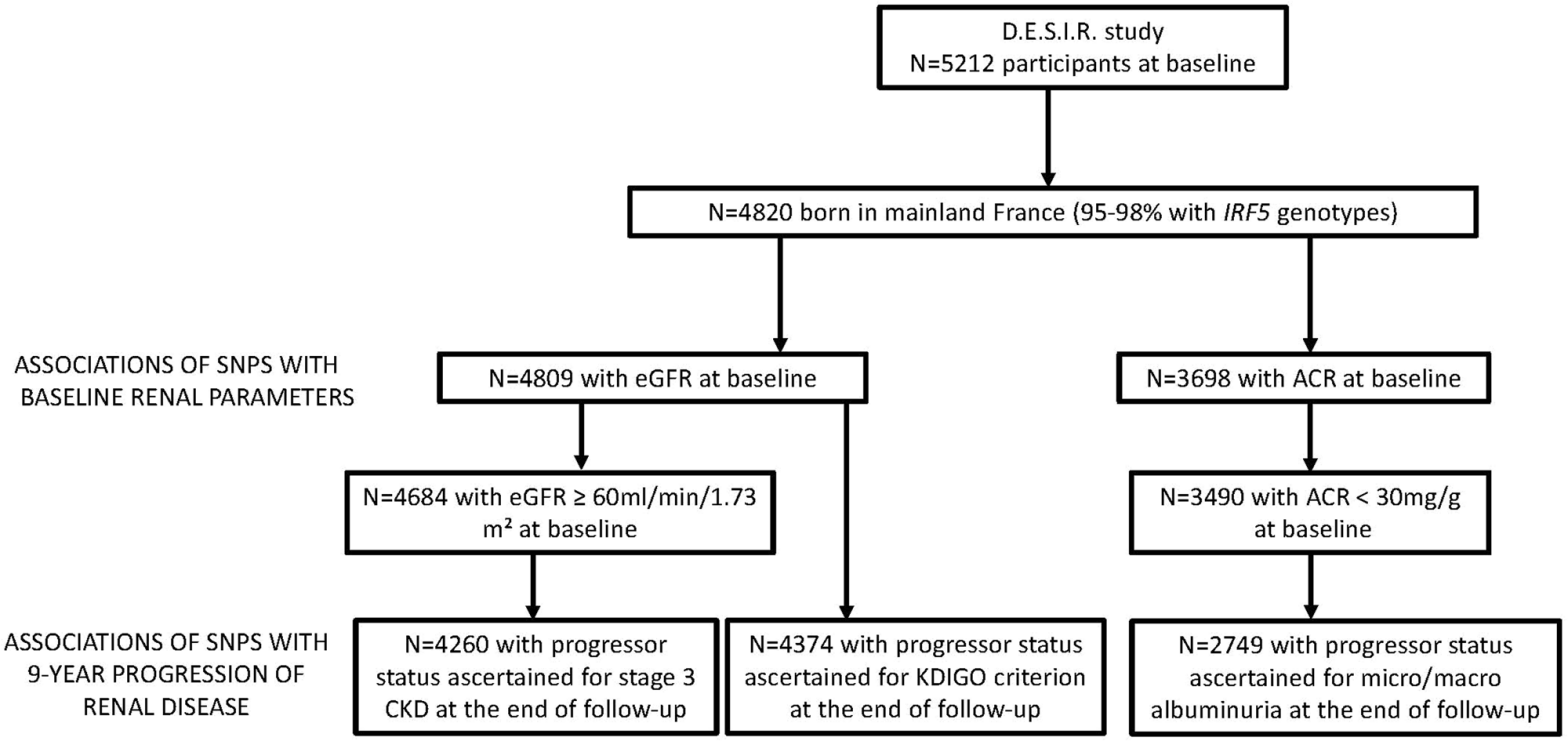
Flow diagram of the study population and design. The D.E.S.I.R. population was used in this study as indicated in the flow diagram to test for cross-sectional and longitudinal associations between *IRF5* polymorphisms and renal phenotypes.

The 9-year incident cases for each criterion were defined in people free of disease by that definition at entry, who developed the disease at some time during the follow-up. Characteristics of participants at baseline by progression of CKD are described in supplementary tables 1–3. They are similar to those we already published in a subset of the D.E.S.I.R. cohort [25]. A flow diagram of the population and the study design is provided in Fig. 1.

### Genotyping

Single nucleotide polymorphisms (SNPs) spanning the whole *IRF5* gene region (chromosome 7q32.1) were selected because they had been found to be associated with human diseases (mainly auto-immune diseases) or as tag SNPs covering 80% of *IRF5* allelic variability with a minor allele frequency >5% in European populations (1000 Genomes Project, GRCh38): rs4731532, rs752637, rs3807306, rs11761199, rs78658945, rs79288514, rs1874328, rs2070197, rs10954213, rs11770589, and rs10954214 (from 5’ to 3’ positions) [26] (Supplementary Table 4). Genotypes were determined by competitive allele-specific PCR genotyping system assays (KASP, LGC Genomics, Hoddesdon, UK). Genotyping success rate was higher than 97%. Genotypes were in Hardy–Weinberg equilibrium (Pearson’s chi-squared test with 1 degree of freedom P > 0.01).

### Statistical analysis

**Fig. 2:**
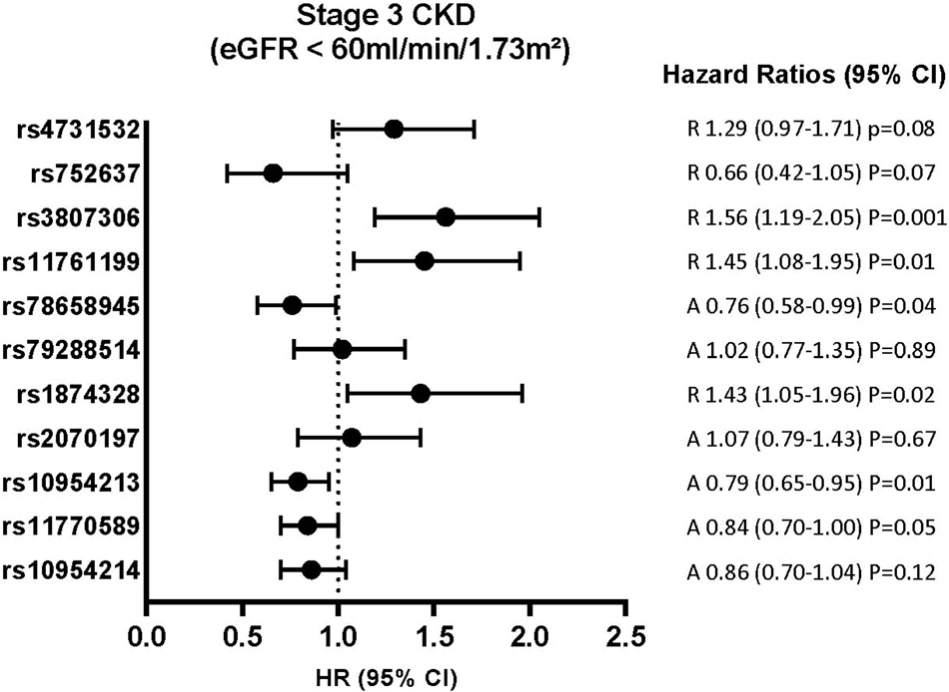
Risk of Stage 3 CKD (eGFR <60 ml/min/1.73 m^2^) incidence during follow-up according to *IRF5* SNPs. Considering the risk for the minor allele: A additive model, D dominant model, R recessive model. Hazard ratio (95% confidence interval) by Cox proportional hazards survival regression model, adjusted for sex, age, BMI, fasting plasma glucose, smoking status at baseline, glycemic status at any time.

**Fig. 3:**
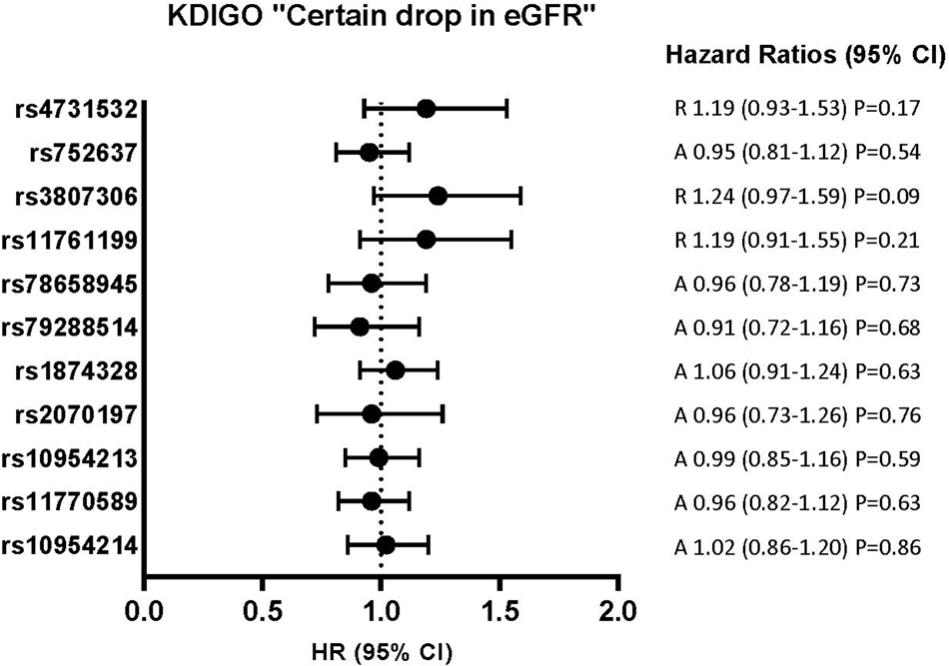
Risk of KDIGO criterion incidence (“Certain drop in eGFR”) during follow-up according to *IRF5* SNPs. Considering the risk for the minor allele: A additive model, D dominant model, R recessive model. Hazard ratio (95% confidence interval) by Cox proportional hazards survival regression model, adjusted for sex, age, BMI, fasting plasma glucose, smoking status at baseline, glycemic status at any time.

**Fig. 4:**
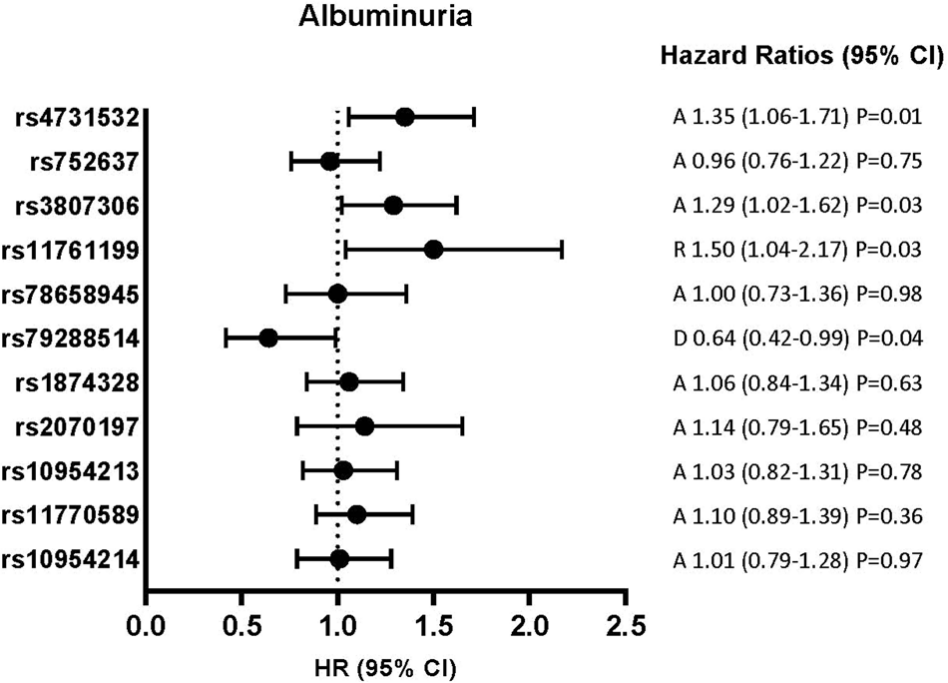
Risk of albuminuria incidence during follow-up according to *IRF5* SNPs. Considering the risk for the minor allele: A additive model, D dominant model, R recessive model. Hazard ratio (95% confidence interval) by Cox proportional hazards survival regression model, adjusted for sex, age, BMI, fasting plasma glucose, smoking status at baseline, glycemic status at any time.

Continuous variables are expressed as mean [standard deviation (SD)] or median (quartiles) and categorical variables as frequencies (percentages). Associations between *IRF5* SNPs with baseline eGFR and ACR (log_e_ transformed) were examined using linear regression analysis and trend tests, after logarithmic transformation for ACR and adjustment for sex and age, then sex, age and BMI. To test for interaction between sex and genotype, we introduced the interaction term in the regression. Because none of the interactions was found statistically significant, we provide the results in the whole population (men and women). Associations between SNPs and incident CKD were first tested by χ^2^ and Cochran trend tests (unadjusted tests), then by Cox proportional hazards survival regression, yielding hazard ratios (HR) with 95% confidence intervals (CI). We first tested the interaction between sex and genotype by a model including only the variables genotype, sex, and genotypeXsex. Because no interaction term was found statistically significant, we performed the analyses in the whole population (men and women). A first adjusted model included as covariates sex, baseline age, body mass index (BMI), fasting plasma glucose, and smoking status as well as the worst glycemic status at any time during follow-up (type 2 diabetes mellitus or impaired fasting glucose [plasma glucose between 6.10 and 6.99 mmol/L]). This first model is presented in Figs. 2–4. A fully adjusted model also included additionally baseline systolic and diastolic blood pressures (SBP and DBP), hypertension (SBP ≥ 140 mmHg or DBP ≥ 90 or treatment for hypertension), use of diuretics or angiotensin-converting enzyme inhibitors (ACEIs) or angiotensin receptor blockers (ARBs), total and high-density lipoprotein (HDL) cholesterol and triglycerides. For kidney function decline according to the first (eGFR <60 ml/ min/1.73 m²) and second (KDIGO criterion) definitions, a supplementary adjustment for baseline eGFR was used for both models. For the incidence of micro/macro albuminuria, a supplementary adjustment for baseline ACR was used. The adjusted tests correspond to the best fitting models of inheritance according to descriptive statistics (additive, dominant or recessive). All models are presented in supplementary Tables 5–7.

*P* < 0.05 was considered to be statistically significant. Since the *IRF5* locus has already been associated with eGFR [20], we considered our study as a replication and did not apply a correction for multiple testing. In our study, for stage 3 CKD incidence, we could detect an OR ≥ 1.30 or ≤0.75 with 80% power for 8/11 of the SNPs tested [minor allele frequency (MAF) ≥ 0.33], and OR ≥ 1.50 or ≤0.60 for all SNPs (codominant model). For CKD defined by the KDIGO “certain drop in eGFR” criterion, we could detect an OR ≥ 1.28 or ≤0.77 with 80% power for 8/11 of the SNPs tested (MAF ≥ 0.33), and OR ≥ 1.43 or ≤ 0.65 for all SNPs. For micro/macroalbuminuria, we could detect an OR ≥ 1.43 or ≤0.68 with 80% power for 8/11 of the SNPs tested (MAF ≥ 0.33), and OR ≥ 1.67 or ≤0.52 for all SNPs.

All statistical analyses were performed with SYSTAT 13 software for Windows (Systat Software, Inc., Chicago, IL 60606, USA). We calculated the power in our sample to detect associations for different ORs by using the Quanto computer program (Gauderman WJ, Morrison JM,QUANTO 1.1: a computer program for power and sample size calculations for genetic-epidemiology studies, 2006; http://hydra.usc.edu/gxe).

## Results

In cross-sectional analyses at baseline, we observed associations of rs10954213 and rs10954214 with eGFR and rs1874328 with ACR (Tables 1 and 2).

**Table 1:** Mean (standard deviation) of eGFR at baseline according to *IRF5* genotypes and P values of trend tests across genotypes in adjusted linear regression analyses.

SNP ID	eGFR (ml/min/1.73 m²) Mean (SD)			P (trend, adjusted for sex, age/sex, age, BMI)
	MM	Mm	mm	
rs4731532	86.8 (13.5)	86.7 (13.8)	86.1 (14.4)	0.44/0.47
rs752637	86.1 (14.0)	86.8 (13.9)	87.5 (13.5)	0.14/0.16
rs3807306	87.1 (13.5)	86.8 (13.9)	85.6 (14.2)	0.09/0.10
rs11761199	87.2 (13.8)	86.6 (13.7)	85.8 (14.3)	0.11/0.12
rs78658945	86.3 (13.9)	87.1 (13.8)	87.8 (12.8)	0.18/0.17
rs79288514	86.7 (13.9)	86.0 (13.6)	88.1 (13.7)	0.26/0.25
rs1874328	87.1 (13.8)	86.5 (13.7)	86.1 (14.1)	0.43/0.41
rs2070197	86.7 (13.8)	86.1 (14.1)	85.7 (14.8)	0.18/0.25
rs10954213	85.9 (13.8)	86.8 (13.9)	87.5 (13.5)	0.04/0.04
rs11770589	86.0 (13.8)	86.6 (13.9)	87.2 (13.8)	0.23/0.21
rs10954214	86.1 (13.8)	86.1 (15.5)	87.9 (13.5)	0.03/0.03

*M* major allele, *m* minor allele.

The D.E.S.I.R. study.

**Table 2:** Mean (quartiles) of urinary albumin/creatinine (ACR) at baseline according to *IRF5* genotypes and P values of trend tests across genotypes in adjusted linear regression analyses.

SNP ID	Urinary ACR (mg/g) Median (25%-75%)			P (trend^a^ adjusted for sex, age/sex, age, BMI)
	MM	Mm	mm	
rs4731532	7.01 (4.80–11.96)	7.24 (4.79–11.59)	7.10 (4.87–12.40)	0.88/0.95
rs752637	7.01 (4.75–11.73)	7.30 (4.87–11.91)	7.06 (4.82–11.79)	0.27/0.30
rs3807306	7.05 (4.83–11.57)	7.15 (4.86–11.71)	7.05 (4.78–12.57)	0.88/0.95
rs11761199	7.14 (4.88–11.70)	7.11 (4.81–11.78)	7.02 (4.75–12.1)	0.54/0.44
rs78658945	7.06 (4.79–11.70)	7.16 (4.83–11.96)	7.99 (4.94–12.99)	0.30/0.33
rs79288514	7.08 (4.80–11.79)	7.11 (4.80–11.56)	7.69 (5.05–12.19)	0.70/0.56
rs1874328	7.21 (4.97–11.81)	7.09 (4.80–11.91)	6.85 (4.49–10.96)	0.01/0.01
rs2070197	7.11 (4.79–11.66)	7.04 (4.95–12.72)	6.97 (4.96–11.14)	0.51/0.64
rs10954213	6.99 (4.68–11.78)	7.23 (4.91–11.80)	7.07 (4.94–11.61)	0.21/0.22
rs11770589	6.85 (4.61–11.48)	7.21 (4.89–11.98)	7.05 (4.98–11.7)	0.06/0.07
rs10954214	7.01 (4.73–11.69)	7.29 (4.93–11.91)	7.11 (4.90–11.98)	0.14/0.15

*M* major allele, *m* minor allele.

^a^Linear regression analysis on log_e_(ACR).

The D.E.S.I.R. study.

After exclusion of people with eGFR <60 ml/min/1.73 m² at baseline, rs3807306, rs11761199, rs78658945, rs1874328, rs10954213 and rs11770589 were associated with the incidence of at least stage 3 CKD at the 9-year follow-up in one or more of the multi-adjusted models (Fig. 2, supplementary Table 5). When adding baseline eGFR in the adjustment covariates, only the associations with rs3807306 and rs1874328 remained statistically significant (supplementary Table 5). For rs3807306, in the model adjusted for age, sex, BMI, fasting plasma glucose, smoking status at baseline, and glycaemic status at any time, the HR (95%CI) was 1.56 (1.19–2.05) P = 0.001, and after additionally adding eGFR in the model: 1.41 (1.07–1.86) P = 0.01. For rs1874328, the HR (95%CI) was 1.43 (1.05–1.96), P = 0.02 and after additionally adding eGFR in the model: 1.38 (1.01–1.88) P = 0.04 (supplementary table 5). Both remained statistically significantly associated after further adjustment in models (supplementary table 5).

Concerning the incidence of CKD defined by the KDIGO “certain drop in eGFR” criterion, we first did not observe any associations in unadjusted tests or by using multi-adjusted models without eGFR. However, rs4731532, rs3807306, and rs11761199 were associated with the incidence of CKD defined by the KDIGO criterion only in the models including baseline eGFR (Fig. 3, supplementary Table 6). For rs3807306, the HR for the model adjusted for sex, age, BMI, fasting plasma glucose, smoking status at baseline, and glycaemic status at any time without eGFR was 1.24 (0.97–1.59) P = 0.09, with eGFR: 1.43 (1.11–1.83) P = 0.005 (supplementary Table 6).

Rs4731532, rs3807306, rs11761199 and rs79288514 were associated with the incidence of albuminuria in multi-adjusted models (Fig. 4, supplementary Table 7). Adding baseline ACR in the models did not modify the strength of the associations. For rs3807306, the HR for the multi-adjusted model (sex, age, BMI, fasting plasma glucose, smoking status at baseline, and glycaemic status at any time) without ACR was 1.29 (1.02–1.62) P = 0.031, with ACR: 1.28 (1.02–1.62) P = 0.034

## Discussion

In this population-based cohort, that was mainly healthy at baseline, *IRF5* genetic variation was associated with eGFR and ACR at baseline, and with the incidence of renal disease assessed by three different criteria.

Most of the associated SNPs are either functional or associated with *IRF5* expression and/or auto-immunity, or in linkage disequilibrium with known functional SNPs. In a study on human systemic lupus erythematosus [27], some variants reside in conserved elements within the 3’ UTR (rs10954214, rs10954213) and the rs10954213 G allele is predicted to disrupt a polyA signal sequence downstream of the stop codon of *IRF5* in the 3’UTR region of exon 9, therefore playing a role in mRNA expression and stability. Other variants associated with *IRF5* expression are located in the exon 1B splice site, such as the rs2004640 [27]. This SNP is in very high linkage disequilibrium with the rs4731532 that we genotyped in our study (r²=0.83 using LDpop online tool on 1000 Genomes European populations [26]). An enhancer variant rs4728142, affecting *IRF5* expression and causal in the association with systemic lupus erythematosus [28], is also in high linkage disequilibrium with some of the SNPS we studied (r²=0.71 both with rs4731532 and rs3807306 in European populations).

The direction of the associations observed in D.E.S.I.R. indicate that impairment in renal function would be associated with an increase in *IRF5* expression, therefore with more inflammation [29].

Our results support the hypothesis that high *IRF5* expression is damaging for the kidney. Since it is also causative in autoimmunity, we wonder whether the associations we observed are a consequence of the renal manifestation of autoimmune diseases such as lupus [30]. Nevertheless, our sample from the D.E.S.I.R. cohort is composed of mainly healthy people. The effect could be direct or could be due to the inflammation associated with *IRF5* overexpression. An epigenome-wide association study for eGFR and ACR showed that DNA methylation at *IRF5* was associated with kidney disease and a Mendelian randomization indicated a causal effect on eGFR [31]. In that study, an increase in methylation at the *IRF5* locus, therefore a lower expression, is accompanied by a gain in eGFR.

Macrophages are present in the kidney in two main forms: resident macrophages [32], or infiltrating macrophages. The latter form derives from circulating monocytes that differentiate into macrophages in situ [33]. Under physiological stress that drives monocyte recruitment to a site of injury, monocytes will tend to differentiate into M1-like proinflammatory macrophages [34]. These cells have been reported to exert a central pathogenic role at the onset of acute kidney injury in animal models. In different models of CKD, M1-like macrophages act in early phases of inflammation [6–8]. IRF5 is a key transcription factor involved in M1-like polarization and in promoting the expression of proinflammatory cytokines [12]. It could be hypothesized that genetic overexpression of *IRF5* could manifest as a basally polarized state in macrophages or result in increased readiness to undergo M1-like polarization. In either of these cases, accelerated or amplified inflammation would predispose to CKD.

Interestingly, while overexpression of *IRF5* has been linked to insulin [14–16, 35, 36] a condition that predisposes to or often coexists with renal disease, our results indicate that the genetic associations with renal disease are independent of insulin resistance, as they remained significant after adjustments for glycemia and diabetes/impaired fasting glycemia status.

Our study has strengths and limitations. The main strength of our study is that we were able to perform a prospective analysis in a cohort with a follow-up of 9 years, in a large general population. This allowed us to show associations with incident CKD, which could not be seen in a previous cross-sectional GWAS meta-analysis [20] showing an association with eGFR, but not with stage 3 CKD. Nevertheless, in D.E.S.I.R., adjusting for baseline eGFR lowered the strength of the associations. As a limitation, we did not measure the true glomerular filtration rate with one of the gold-standard methods, as they are not easily applicable to large cohort studies. Instead, we used estimations based on plasma creatinine. The SNP highlighted in the large meta-analysis on eGFR [20], rs3757387, could not be genotyped in our study. Nevertheless, this SNP is in high linkage disequilibrium with rs3807306 (r²=0.78 using LDpop online tool on 1000 Genomes European populations [26]), the SNP most highly associated with renal function of all the SNPs we studied. Our study included people of European descent and our conclusions may not apply to people from other ethnic backgrounds. However, it is noteworthy that the reported association between *IRF5* locus and eGFR was observed in a trans-ancestry study, including individuals from European, East Asian, African-American South Asian and Hispanic origins [20]. Finally, the observational design of our study does not allow us to conclude a causal relationship between *IRF5* genetic variation and CKD, but rather allows us to raise some hypotheses.

In conclusion, in a cohort from the general population, *IRF5* genetic polymorphisms were associated with renal function at baseline and at follow-up. This relationship may be mediated by macrophage-dependent inflammation at the kidney level.

### Supplementary information

Supplementary tables 1-4

Supplementary tables 5-7

## Data Availability

The data underlying this article are available in Figshare, 10.6084/m9.figshare.22559947.
