# Supplementary material for: Genetic variants of interferon-response factor 5 are associated with the incidence of chronic kidney disease: the D.E.S.I.R. study
Source: Genes Immun. 2023 Nov 17;24(6):303–8. doi: 10.1038/s41435-023-00229-4 (PMC10721545; doi:10.1038/s41435-023-00229-4)
Supplement: Supplementary file 1 — Supplementary tables 1-4 [file 41435_2023_229_MOESM1_ESM.docx]

**Supplementary tables 1-4**

**Supplementary table 1**

**Characteristics of participants at baseline by progression of CKD during follow-up (subjects with eGFR ≥ 60 ml/min/1.73 m² at baseline, n=4260). The D.E.S.I.R Study**

**Supplementary table 2**

**Characteristics of participants at baseline by progression of KDIGO during follow-up (n=4374). The D.E.S.I.R Study**

**Supplementary table 3**

**Characteristics of participants at baseline by progression of albuminuria during follow-up (subjects with ACR < 30 mg/g at baseline, n=2749). The D.E.S.I.R Study**

**Supplementary table 4**

**IRF5 SNPs properties**

**ABBREVIATIONS**

ACE-I: angiotensin converting enzyme inhibitor; ACR: albumin creatinine ratio; ARB: angiotensin 2 receptor blocker; BMI, body mass index; DBP, diastolic blood pressure; T2DM: type 2 diabetes mellitus; eGFR: estimated glomerular filtration rate; HOMA2-IR: insulin resistance index; IFG: impaired fasting glucose; HDL, high-density lipoprotein; LDL, low-density lipoprotein; NFG: normal fasting glucose; SBP, systolic blood pressure

**Supplementary Table 1.**

**Characteristics of participants at baseline by progression of CKD during follow-up (subjects with eGFR ≥ 60 ml/min/1.73 m² at baseline, n=4260). The D.E.S.I.R Study**

|  |  |  | P value |
| --- | --- | --- | --- |
|  | Stage 3 CKD (eGFR < 60 ml/min/1.73 m²) | |  |
|  | **Non progressors** | **progressors** |  |
| **N** | 4005 | 255 |  |
| **Age, years** | 46.2 (9.8) | 56.2 (7.6) | <0.001 |
| **Men, %** | 51 | 35 | <0.001 |
| **BMI, kg/m²** | 24.6 (3.7) | 25.2 (4.0) | 0.009 |
| **Waist, cm** | 83.1 (11.6) | 83.9 (12.0) | 0.26 |
| **SBP, mmHg** | 131 (15) | 136 (17) | <0.001 |
| **DBP, mmHg** | 80 (9) | 81 (10) | 0.004 |
| **Arterial hypertension, %** | 33 | 52 | <0.001 |
| **Anti-hypertensive treatment, %** | 7.3 | 22.0 | <0.001 |
| **Use of ACE-I or ARB, %** | 3.1 | 9.0 | <0.001 |
| **Use of diuretics, %** | 2.7 | 7.8 | <0.001 |
| **eGFR, ml/min/1.73m²** | 88 (13) | 72 (9) | <0.001 |
| **Urinary ACR, mg/g*** | 7.1 (4.8-11.6) | 7.3 (4.7-12.9) | 0.08 |
| **Current smoker, %** | 22 | 10 | <0.001 |
| **Glycemic status: NFG/IFG/T2DM, %** | 91/6.5/2.3 | 90/6.7/3.1 | 0.72 |
| **Fasting plasma glucose, mmol/l** | 5.3 (0.8) | 5.4 (1.0) | 0.40 |
| **Plasma cholesterol, mmol/l** | 5.7 (1.0) | 6.0 (0.9) | <0.001 |
| **LDL cholesterol, mmol/l** | 3.6 (0.9) | 3.8 (0.8) | <0.001 |
| **HDL cholesterol, mmol/l** | 1.6 (0.4) | 1.7 (0.4) | 0.07 |
| **Triglycerides, mmol/l** | 0.95 (0.67-1.37) | 1.07 (0.78-1.37) | 0.06 |
| **HOMA2-IR** | 0.9 (0.7-1.3) | 0.9 (0.7-1.2) | 0.92 |

Data expressed as mean (SD) or percent, urinary albumin creatinine ratio, triglycerides and HOMA2-IR, expressed as median (quartiles).

P values are from Pearson's chi-squared tests (qualitative variables) or Student’s t-test (quantitative variables), with log-transformed data when appropriate.

* With ACR: non progressors n=3075, progressors n=207

**Supplementary Table 2.**

**Characteristics of participants at baseline by progression of KDIGO during follow-up (n=4374). The D.E.S.I.R Study**

|  |  |  | P value |
| --- | --- | --- | --- |
|  | KDIGO | |  |
|  | **Non progressors** | **progressors** |  |
| **N** | 4041 | 333 |  |
| **Age, years** | 47.0 (10.0) | 48..2 (10.2) | 0.03 |
| **Men, %** | 50 | 39 | <0.001 |
| **BMI, kg/m²** | 24.7 (3.7) | 24.5 (3.7) | 0.28 |
| **Waist, cm** | 83.4 (11.6) | 81.6 (11.7) | 0.007 |
| **SBP, mmHg** | 131 (15) | 131 (17) | 0.81 |
| **DBP, mmHg** | 80 (9) | 80 (10) | 0.69 |
| **Arterial hypertension, %** | 35 | 35 | 0.85 |
| **Anti-hypertensive treatment, %** | 8.6 | 10.8 | 0.16 |
| **Use of ACE-I or ARB, %** | 3.4 | 5.4 | 0.06 |
| **Use of diuretics, %** | 3.4 | 3.3 | 0.90 |
| **eGFR, ml/min/1.73m²** | 86 (14) | 95 (14) | <0.001 |
| **Urinary ACR, mg/g*** | 7.1 (4.8-11.7) | 7.3 (5.2-13.2) | 0.11 |
| **Current smoker, %** | 21 | 19 | 0.42 |
| **Glycemic status: NFG/IFG/T2DM, %** | 91/6.7/2.5 | 92/5.1/2.4 | 0.51 |
| **Fasting plasma glucose, mmol/l** | 5.4 (0.8) | 5.3 (0.8) | 0.11 |
| **Plasma cholesterol, mmol/l** | 5.7 (1.0) | 5.7 (1.0) | 0.92 |
| **LDL cholesterol, mmol/l** | 3.6 (0.9) | 3.6 (0.9) | 0.86 |
| **HDL cholesterol, mmol/l** | 1.6 (0.4) | 1.7 (0.4) | 0.14 |
| **Triglycerides, mmol/l** | 0.96 (0.68-1.38) | 0.97 (0.65-1.33) | 0.44 |
| **HOMA2-IR** | 0.9 (0.7-1.3) | 0.9 (0.7-1.2) | 0.15 |

Data expressed as mean (SD) or percent, urinary albumin creatinine ratio, triglycerides and HOMA2-IR, expressed as median (quartiles).

P values are from Pearson's chi-squared tests (qualitative variables) or Student’s t-test (quantitative variables), with log-transformed data when appropriate.

*With ACR: non progressors n=3108, progressors n=260

**Supplementary Table 3.**

**Characteristics of participants at baseline by progression of albuminuria during follow-up (subjects with ACR < 30 mg/g at baseline, n=2749). The D.E.S.I.R Study**

|  |  |  | P value |
| --- | --- | --- | --- |
|  | Albuminuria (ACR ≥ 30mg/g) | |  |
|  | **Non progressors** | **progressors** |  |
| **N** | 2600 | 149 |  |
| **Age, years** | 47.2 (9..8) | 52.0 (9.5) | <0.001 |
| **Men, %** | 54 | 43 | 0.009 |
| **BMI, kg/m²** | 24.6 (3.5) | 26.2 (4.5) | <0.001 |
| **Waist, cm** | 83.3 (11.2) | 87.3 (13.4) | <0.001 |
| **SBP, mmHg** | 131 (15) | 137 (18) | <0.001 |
| **DBP, mmHg** | 80 (9) | 84 (10) | <0.001 |
| **Arterial hypertension, %** | 34 | 54 | <0.001 |
| **Anti-hypertensive treatment, %** | 7.7 | 23.5 | <0.001 |
| **Use of ACE-I or ARB, %** | 3.2 | 10.7 | <0.001 |
| **Use of diuretics, %** | 2.7 | 14.8 | <0.001 |
| **eGFR, ml/min/1.73m²** | 86 (13) | 80 (14) | <0.001 |
| **Urinary ACR, mg/g** | 6.6 (4.6-10.0) | 10.5 (6.2-16.8) | <0.001 |
| **Current smoker, %** | 19 | 17 | 0.55 |
| **Glycemic status: NFG/IFG/T2DM, %** | 92/6.0/2.0 | 82/11.4/6.7 | 0.001 |
| **Fasting plasma glucose, mmol/l** | 5.3 (0.7) | 5.6 (1.3) | <0.001 |
| **Plasma cholesterol, mmol/l** | 5.7 (1.0) | 5.8 (1.0) | 0.16 |
| **LDL cholesterol, mmol/l** | 3.6 (0.9) | 3.6 (0.9) | 0.90 |
| **HDL cholesterol, mmol/l** | 1.6 (0.4) | 1.7 (0.4) | 0.73 |
| **Triglycerides, mmol/l** | 0.96 (0.67-1.37) | 1.08 (0.75-1.61) | 0.004 |
| **HOMA2-IR** | 0.9 (0.7-1.2) | 1.1 (0.7-1.5) | 0.002 |

Data expressed as mean (SD) or percent, urinary albumin creatinine ratio, triglycerides and HOMA2-IR, expressed as median (quartiles).

P values are from Pearson's chi-squared tests (qualitative variables) or Student’s t-test (quantitative variables), with log-transformed data when appropriate.

**Supplementary table 4**

**IRF5 SNPs properties**

| **SNP ID** | **Position**  **GRCh38** | **Alleles Major/minor** | **MAF D.E.S.I.R.** |
| --- | --- | --- | --- |
| **rs4731532** | 7:128932712 | G/A | 0.499 |
| **rs752637** | 7:128939366 | G/A | 0.365 |
| **rs3807306** | 7:128940626 | C/A | 0.482 |
| **rs11761199** | 7:128941781 | A/G | 0.436 |
| **rs78658945** | 7:128941829 | G/A | 0.165 |
| **rs79288514** | 7:128942604 | C/T | 0.122 |
| **rs1874328** | 7:128945050 | T/C | 0.390 |
| **rs2070197** | 7:128948946 | T/C | 0.097 |
| **rs10954213** | 7:128949373 | A/G | 0.382 |
| **rs11770589** | 7:128949434 | A/G | 0.478 |
| **rs10954214** | 7:128949579 | T/C | 0.334 |

MAF=minor allele frequency
