# Supplementary material for: Genetic variants of interferon-response factor 5 are associated with the incidence of chronic kidney disease: the D.E.S.I.R. study
Source: Genes Immun. 2023 Nov 17;24(6):303–8. doi: 10.1038/s41435-023-00229-4 (PMC10721545; doi:10.1038/s41435-023-00229-4)
Supplement: Supplementary file 2 — Supplementary tables 5-7 [file 41435_2023_229_MOESM2_ESM.docx]

**Supplementary tables 5-7**

**Supplementary Table 5**

**Genotype frequencies of IRF5 polymorphisms according to the incidence of Stage 3 CKD (eGFR < 60 ml/min/1.73 m²). The D.E.S.I.R. study**

**Supplementary Table 6**

**Genotype frequencies of IRF5 polymorphisms according to the incidence of CKD KDIGO definition. The D.E.S.I.R. study**

**Supplementary Table 7**

**Genotype frequencies of IRF5 polymorphisms according to the incidence of albuminuria. The D.E.S.I.R. study**

**Supplementary Table 5**

**Genotype frequencies of *IRF5* polymorphisms according to the incidence of Stage 3 CKD (eGFR < 60 ml/min/1.73 m²). The D.E.S.I.R. study**

| **SNP** | **Genotype** | **Controls** | **Cases** | **Incidence (%)** | **P chi² 2df/trend/ dominant/recessive** | **HR1^a^** | **P** | **HR2^b^** | **P** |
| --- | --- | --- | --- | --- | --- | --- | --- | --- | --- |
|  |  |  |  |  |  |  |  |  |  |
| rs4731532 | GG  GA  AA | 919 (24.2%)  1 972 (52.0%)  904 (23.8%) | 52 (21.5%)  119 (49.2%)  71 (29.3%) | 5.4  5.7  7.3 | 0.14/0.08/0.34/0.05 | R 1.29 (0.97-1.71)  R* 1.24 (0.93-1.65) | 0.08  0.13 | 1.28 (0.96-1.71)  1.23 (0.92-1.64) | 0.09  0.16 |
|  |  |  |  |  |  |  |  |  |  |
| rs752637 | GG  GA  AA | 1 540 (40.0%)  1 789 (46.5%)  517 (13.4%) | 111 (45.1%)  114 (46.3%)  21 (8.5%) | 6.7  6.0  3.9 | 0.06/0.03/0.12/0.03 | R 0.66 (0.42-1.05)  R* 0.75 (0.47-1.19) | 0.07  0.21 | 0.67 (0.42-1.06)  0.76 (0.48-1.21) | 0.08  0.24 |
|  |  |  |  |  |  |  |  |  |  |
| rs3807306 | CC  CA  AA | 1 071 (27.3%)  1 961 (50.1%)  884 (22.6%) | 56 (22.4%)  113 (45.2%)  81 (32.4%) | 5.0  5.4  8.4 | 0.002/0.002/0.09/0.0004 | R 1.56 (1.19-2.05)  R* 1.41 (1.07-1.86) | 0.001  0.01 | 1.55 (1.18-2.04)  1.40 (1.06-1.85) | 0.002  0.02 |
|  |  |  |  |  |  |  |  |  |  |
| rs11761199 | AA  AG  GG | 1 270 (32.4%)  1 918 (48.9%)  731 (18.7%) | 72 (28.8%)  114 (45.6%)  64 (25.6%) | 5.4  5.6  8.1 | 0.02/0.03/0.24/0.007 | R 1.45 (1.08-1.95)  R* 1.33 (0.99-1.78 | 0.01  0.05 | 1.40 (1.04-1.89)  1.29 (0.96-1.73) | 0.02  0.09 |
|  |  |  |  |  |  |  |  |  |  |
| rs78658945 | GG  GA  AA | 2 711 (68.8%)  1 127 (28.6%)  100 (2.5%) | 192 (76.2%)  55 (21.8%)  5 (2.0%) | 6.6  4.7  4.8 | 0.05/0.009/0.01/0.58 | A 0.76 (0.58-0.99)  A* 0.82 (0.63-1.07) | 0.04  0.14 | 0.74 (0.57-0.97)  0.82 (0.62-1.07) | 0.03  0.13 |
|  |  |  |  |  |  |  |  |  |  |
| rs79288514 | CC  CT  TT | 3 038 (77.3%)  834 (21.2%)  58 (1.5%) | 191 (76.4%)  57 (22.8%)  2 (0.8%) | 5.9  6.4  3.3 | 0.59/0.79/0.74/0.38 | A 1.02 (0.77-1.35)  A* 1.03 (0.78-1.36) | 0.89  0.83 | 1.02 (0.77-1.35)  1.03 (0.78-1.36) | 0.91  0.83 |
|  |  |  |  |  |  |  |  |  |  |
| rs1874328 | TT  TC  CC | 1 443 (37.7%)  1 815(47.4%)  573 (15.0%) | 82 (33.9%)  107 (44.2%)  53 (21.9%) | 5.4  5.6  8.5 | 0.01/0.04/0.24/0.004 | R 1.43 (1.05-1.96)  R* 1.38 (1.01-1.88) | 0.02  0.04 | 1.44 (1.05-1.97)  1.38 (1.00-1.89) | 0.02  0.04 |
|  |  |  |  |  |  |  |  |  |  |
| rs2070197 | TT  TC  CC | 3 221 (81.9%)  669 (17.0%)  42 (1.1%) | 204 (81.0%)  45 (17.9%)  3 (1.2%) | 6.0  6.3  6.7 | 0.92/0.70/0.70/0.86 | A 1.07 (0.79-1.43)  A* 1.0 (0.74-1.35) | 0.67  0.99 | 1.06 (0.79-1.43)  0.98 (0.73-1.33) | 0.68  0.90 |
|  |  |  |  |  |  |  |  |  |  |
|  |  |  |  |  |  |  |  |  |  |
| **Supplementary Table 5 continued** | | |  |  |  |  |  |  |  |
| **SNP** | **Genotype** | **Controls** | **Cases** | **Incidence (%)** | **P chi² 2df/trend/ dominant/recessive** | **HR1^a^** | **P** | **HR2^b^** | **P** |
| rs10954213 | AA  AG  GG | 1 464 (37.4%)  1 861 (47.5%)  590 (15.1%) | 112 (44.8%)  114 (45.6%)  24 (9.6%) | 7.1  5.8  3.9 | 0.02/0.01/0.02/0.02 | A 0.79 (0.65-0.95)  A* 0.84 (0.69-1.02) | 0.01  0.08 | 0.79 (0.65-0.96)  0.85 (0.69-1.03) | 0.01  0.09 |
|  |  |  |  |  |  |  |  |  |  |
| rs11770589 | AA  AG  GG | 1 046 (27.3%)  1 872 (48.9%)  914 (23.9%) | 85 (34.3%)  116 (46.8%)  47 (19.0%) | 7.5  5.8  4.9 | 0.04/0.01/0.02/0.08 | A 0.84 (0.70-1.00)  A* 0.86 (0.72-1.03) | 0.05  0.10 | 0.83 (0.70-1.00)  0.86 (0.71-1.03) | 0.05  0.10 |
|  |  |  |  |  |  |  |  |  |  |
| rs10954214 | TT  TC  CC | 1 679 (43.6%)  1 735 (45.1%)  436 (11.3%) | 119 (48.2%)  109 (44.1%)  19 (7.7%) | 6.6  5.9  4.2 | 0.14/0.07/0.16/0.08 | A 0.86 (0.70-1.04)  A* 0.91 (0.74-1.12) | 0.12  0.36 | 0.86 (0.70-1.05)  0.91 (0.75-1.12) | 0.12  0.38 |

Considering the risk for the minor allele: A= additive model, D = dominant model, R= recessive model

^a^ HR1=Hazard ratio (95% confidence interval) by Cox proportional hazards survival regression, adjusted for sex, age, BMI, fasting plasma glucose, smoking status at baseline, and glycemic status at any time. *=same model, plus baseline eGFR

^b^ HR2=Hazard ratio (95% confidence interval), same model as HR1 with additional adjustment for systolic and diastolic blood pressures, hypertension, use of diuretics, use of angiotensin-converting enzyme inhibitors or angiotensin receptor blockers, total and HDL cholesterol, triglycerides at baseline. * same model plus baseline eGFR

**Supplementary Table 6**

**Genotype frequencies of *IRF5* polymorphisms according to the incidence of CKD KDIGO definition. The D.E.S.I.R. study**

| **SNP** | **Genotype** | **Controls** | **Cases** | | **Incidence (%)** | | **P chi² 2df/trend/ dominant/recessive** | | **HR1^a^** | | **P** | | **HR2^b^** | | **P** | |
| --- | --- | --- | --- | --- | --- | --- | --- | --- | --- | --- | --- | --- | --- | --- | --- | --- |
|  |  |  |  | |  | |  | |  | |  | |  | |  | |
| rs4731532 | GG  GA  AA | 917 (23.9%)  1 993 (52.1%)  919 (24.0%) | 80 (25.4%)  150 (47.6%)  85 (27.0%) | | 8.0  7.0  8.5 | | 0.30/0.72/0.56/0.24 | | R 1.19 (0.93-1.53)  R* 1.32 (1.03-1.69) | | 0.17  0.03 | | 1.17 (0.91-1.51)  1.29 (1.00-1.66) | | 0.21  0.05 | |
|  |  |  |  | |  | |  | |  | |  | |  | |  | |
| rs752637 | GG  GA  AA | 1 565 (40.3%)  1 805 (46.5%)  513 (13.2%) | 129 (41.0%)  149 (47.3%)  37 (11.7%) | | 7.6  7.6  6.7 | | 0.76/0.64/0.82/0.46 | | A 0.95 (0.81-1.12)  A* 0.89 (0.76-1.06 | | 0.54  0.20 | | 0.96 (0.81-1.13)  0.91 (0.77-1.07) | | 0.64  0.26 | |
|  |  |  |  | |  | |  | |  | |  | |  | |  | |
| rs3807306 | CC  CA  AA | 1 064 (26.9%)  1 979 (50.1%)  909 (23.0%) | 91 (28.1%)  148 (45.7%)  85 (26.2%) | | 7.9  7.0  8.6 | | 0.26/0.64/0.65/0.19 | | R 1.24 (0.97-1.59)  R* 1.43 (1.11-1.83) | | 0.09  0.005 | | 1.23 (0.95-1.57)  1.41 (1.09-1.81) | | 0.11  0.008 | |
|  |  |  |  | |  | |  | |  | |  | |  | |  | |
| rs11761199 | AA  AG  GG | 1 266 (32.0%)  1 938 (49.0%)  750 (19.0%) | 112 (34.5%)  146 (44.9%)  67 (20.6%) | | 8.1  7.0  8.2 | | 0.37/0.79/0.37/0.47 | | R 1.19 (0.91-1.55)  R* 1.33 (1.01-1.74) | | 0.21  0.04 | | 1.17 (0.89-1.54)  1.30 (0.99-1.72) | | 0.25  0.06 | |
|  |  |  |  | |  | |  | |  | |  | |  | |  | |
| rs78658945 | GG  GA  AA | 2 748 (69.1%)  1 126 (28.3%)  100 (2.5%) | 228 (69.7%)  92 (28.1%)  7 (2.1%) | | 7.7  7.6  6.5 | | 0.91/0.80/0.83/0.68 | | A 0.96 (0.78-1.19)  A* 0.89 (0.72-1.11) | | 0.73  0.31 | | 0.97 (0.78-1.19)  0.90 (0.72-1.11) | | 0.75  0.33 | |
|  |  |  |  | |  | |  | |  | |  | |  | |  | |
| rs79288514 | CC  CT  TT | 3 057 (77.1%)  850 (21.4%)  56 (1.4%) | 253 (77.6%)  68 (20.9%)  5 (1.5%) | | 7.6  7.4  8.2 | | 0.96/0.86/0.85/0.86 | | A 0.91 (0.72-1.16)  A* 0.92 (0.73-1.17) | | 0.68  0.32 | | 0.89 (0.70-1.14)  0.90 (0.71-1.14) | | 0.67  0.39 | |
|  |  |  |  | |  | |  | |  | |  | |  | |  | |
| rs1874328 | TT  TC  CC | 1 458 (37.7%)  1 820 (47.1%)  588 (15.2%) | 115 (36.7%)  144 (46.0%)  54 (17.3%) | | 7.3  7.3  8.4 | | 0.63/0.51/0.73/0.34 | | A 1.06 (0.91-1.24)  A* 1.10 (0.94-1.29) | | 0.63  0.23 | | 1.06 (0.90-1.24)  1.10 (0.93-1.29) | | 0.47  0.27 | |
|  |  |  |  | |  | |  | |  | |  | |  | |  | |
| rs2070197 | TT  TC  CC | 3 240 (81.6%)  684 (17.2%)  45 (1.1%) | 273 (83.7%)  51 (15.6%)  2 (0.6%) | | 7.8  6.9  4.3 | | 0.51/0.31/0.34/0.39 | | A 0.96 (0.73-1.26)  A* 1.05 (0.79-1.38) | | 0.76  0.75 | | 0.95 (0.72-1.26)  1.05 (0.79-1.39) | | 0.74  0.74 | |
|  |  |  |  | |  | |  | |  | |  | |  | |  | |
|  |  |  |  | |  | |  | |  | |  | |  | |  | |
|  |  |  |  | |  | |  | |  | |  | |  | |  | |
| **Supplementary Table 6 continued** | | | |  | |  | |  | |  | |  | |  | |  |
| **SNP** | **Genotype** | **Controls** | **Cases** | | **Incidence (%)** | | **P chi² 2df/trend/ dominant/recessive** | | **HR1^a^** | | **P** | | **HR2^b^** | | **P** | |
| rs10954213 | AA  AG  GG | 1 493 (37.8%)  1 872 (47.4%)  583 (14.8%) | 124 (38.2%)  152 (46.7%)  49 (15.1%) | | 7.7  7.5  7.8 | | 0.97/0.98/0.90/0.88 | | A 0.99 (0.85-1.16)  A* 0.93 (0.79-1.09) | | 0.59  0.36 | | 1.01 (0.86-1.18)  0.94 (0.80-1.11) | | 0.95  0.47 | |
|  |  |  |  | |  | |  | |  | |  | |  | |  | |
| rs11770589 | AA  AG  GG | 1 064 (27.5%)  1 890 (48.8%)  916 (23.7%) | 95 (30.0%)  150 (47.3%)  72 (22.7%) | | 8.2  7.4  7.3 | | 0.64/0.41/0.34/0.70 | | A 0.96 (0.82-1.12)  A* 0.93 (0.79-1.08) | | 0.63  0.33 | | 0.97 (0.83-1.14)  0.94 (0.80-1.10) | | 0.73  0.45 | |
|  |  |  |  | |  | |  | |  | |  | |  | |  | |
| rs10954214 | TT  TC  CC | 1 710 (44.0%)  1 746 (44.9%)  431 (11.1%) | 136 (42.8%)  147 (46.2%)  35 (11.0%) | | 7.4  7.8  7.5 | | 0.90/0.73/0.67/0.96 | | A 1.02 (0.86-1.20)  A* 0.96 (0.80-1.12) | | 0.86  0.51 | | 1.02 (0.87-1.21)  0.96 (0.81-1.13) | | 0.78  0.59 | |

Considering the risk for the minor allele: A= additive model, D = dominant model, R= recessive model

^a^ HR1=Hazard ratio (95% confidence interval) by Cox proportional hazards survival regression, adjusted for sex, age, BMI, fasting plasma glucose, smoking status at baseline, and glycemic status at any time. *=same model, plus baseline eGFR

^b^ HR2=Hazard ratio (95% confidence interval), same model as HR1 with additional adjustment for systolic and diastolic blood pressures, hypertension, use of diuretics, use of angiotensin-converting enzyme inhibitors or angiotensin receptor blockers, total and HDL cholesterol, triglycerides at baseline. * same model plus baseline eGFR

**Supplementary Table 7**

**Genotype frequencies of *IRF5* polymorphisms according to the incidence of albuminuria. The D.E.S.I.R. study**

| **SNP** | **Genotype** | **Controls** | **Cases** | | **Incidence (%)** | | **P chi² 2df/trend/ dominant/recessive** | | **HR1^a^** | | **P** | | **HR2^b^** | | **P** | |
| --- | --- | --- | --- | --- | --- | --- | --- | --- | --- | --- | --- | --- | --- | --- | --- | --- |
|  |  |  |  | |  | |  | |  | |  | |  | |  | |
| rs4731532 | GG  GA  AA | 615 (24.8%)  1 262 (51.0%)  599 (24.2%) | 25 (17.5%)  70 (49.0%)  48 (33.6%) | | 3.9  5.3  7.4 | | 0.02/0.005/0.05/0.01 | | A 1.35 (1.06-1.71)  A* 1.34 (1.05-1.69) | | 0.01  0.02 | | 1.31 (1.03-1.67)  1.29 (1.01-1.65) | | 0.03  0.04 | |
|  |  |  |  | |  | |  | |  | |  | |  | |  | |
| rs752637 | GG  GA  AA | 1 021 (40.9%)  1 133 (45.4%)  343 (13.7%) | 63 (43.8%)  63 (43.8%)  18 (12.5%) | | 5.8  5.3  5.0 | | 0.78/0.48/0.50/0.67 | | A 0.96 (0.76-1.22)  A* 0.96 (0.75-1.22) | | 0.75  0.71 | | 0.98 (0.76-1.25)  0.97 (0.76-1.25) | | 0.85  0.83 | |
|  |  |  |  | |  | |  | |  | |  | |  | |  | |
| rs3807306 | CC  CA  AA | 708 (27.9%)  1 249 (49.2%)  581 (22.9%) | 30 (20.4%)  74 (50.3%)  43 (29.3%) | | 4.1  5.6  6.9 | | 0.07/0.02/0.05/0.08 | | A 1.29 (1.02-1.62)  A* 1.28 (1.02-1.62) | | 0.03  0.03 | | 1.25 (0.99-1.58)  1.23 (0.97-1.56) | | 0.07  0.08 | |
|  |  |  |  | |  | |  | |  | |  | |  | |  | |
| rs11761199 | AA  AG  GG | 838 (32.9%)  1 223 (48.1%)  483 (19.0%) | 40 (27.2%)  69 (46.9%)  38 (25.9%) | | 4.6  5.3  7.3 | | 0.09/0.04/0.15/0.04 | | R 1.50 (1.04-2.17)  R* 1.51 (1.04-2.18) | | 0.03  0.03 | | 1.51 (1.04-2.21)  1.53 (1.04-2.23) | | 0.03  0.03 | |
|  |  |  |  | |  | |  | |  | |  | |  | |  | |
| rs78658945 | GG  GA  AA | 1 775 (69.4%)  715 (28.0%)  66 (2.6%) | 106 (71.6%)  37 (25.0%)  5 (3.4%) | | 5.6  4.9  7.0 | | 0.65/0.63/0.58/0.56 | | A 1.00 (0.73-1.36)  A* 0.95 (0.70-1.30) | | 0.98  0.76 | | 0.97 (0.70-1.34)  0.93 (0.68-1.28) | | 0.85  0.65 | |
|  |  |  |  | |  | |  | |  | |  | |  | |  | |
| rs79288514 | CC  CT  TT | 1 950 (76.6%)  564 (22.2%)  32 (1.3%) | 121 (82.3%)  24 (16.3%)  2 (1.4%) | | 5.8  4.1  5.9 | | 0.25/0.09/0.11/0.91 | | D 0.64 (0.42-0.99)  D* 0.65 (0.42-0.99) | | 0.04  0.05 | | 0.61 (0.40-0.95)  0.61 (0.39-0.95) | | 0.03  0.03 | |
|  |  |  |  | |  | |  | |  | |  | |  | |  | |
| rs1874328 | TT  TC  CC | 941 (37.8%)  1 145 (46.0%)  402 (16.2%) | 52 (36.6%)  65 (45.8%)  25 (17.6%) | | 5.2  5.4  5.9 | | 0.89/0.68/0.77/0.65 | | A 1.06 (0.84-1.34)  A* 1.10 (0.87-1.39) | | 0.63  0.44 | | 1.06 (0.84-1.35)  1.09 (0.86-1.39) | | 0.62  0.47 | |
|  |  |  |  | |  | |  | |  | |  | |  | |  | |
| rs2070197 | TT | 2 110 (82.6%)  413 (16.2%)  33 (1.3%) | 116 (78.9%)  30 (20.4%)  1 (0.7%) | | 5.2  6.8  2.9 | | 0.34/0.31/0.26/0.52 | | A 1.14 (0.79-1.65)  A* 1.14 (0.79-1.64) | | 0.48  0.49 | | 1.18 (0.82-1.71)  1.17 (0.80-1.69) | | 0.38  0.42 | |
|  | TC |  |  |  |  |  |  |  |  |  |  |  |  |  |  |  |
|  | CC |  |  |  |  |  |  |  |  |  |  |  |  |  |  |  |
|  |  |  |  | |  | |  | |  | |  | |  | |  | |
| **Supplementary Table 7 continued** | | | |  | |  | |  | |  | |  | |  | |  |
| **SNP** | **Genotype** | **Controls** | **Cases** | | **Incidence (%)** | | **P chi² 2df/trend/ dominant/recessive** | | **HR1^a^** | | **P** | | **HR2^b^** | | **P** | |
| rs10954213 | AA | 974 (38.4%)  1 177 (46.4%)  386 (15.2%) | 60 (40.8%)  61 (41.5%)  26 (17.7%) | | 5.8 | | 0.48/0.91/0.56/0.42 | | A 1.03 (0.82-1.31)  A* 1.02 (0.81-1.29) | | 0.78  0.87 | | 1.04 (0.82-1.32)  1.03 (0.81-1.31) | | 0.76  0.80 | |
|  | AG |  |  |  | 4.9 | |  |  |  |  |  |  |  |  |  |  |
|  | GG |  |  |  | 6.3 | |  |  |  |  |  |  |  |  |  |  |
|  |  |  |  | |  | |  | |  | |  | |  | |  | |
| rs11770589 | AA | 707 (28.5%)  1 190 (47.9%)  587 (23.6%) | 41 (28.5%)  63 (43.8%)  40 (27.8%) | | 5.5  5.0  6.4 | | 0.48/0.53/1.00/0.26 | | A 1.10 (0.89-1.39)  A* 1.09 (0.87-1.37) | | 0.36  0.44 | | 1.14 (0.90-1.44)  1.12 (0.89-1.42) | | 0.28  0.33 | |
|  | AG |  |  |  |  |  |  |  |  |  |  |  |  |  |  |  |
|  | GG |  |  |  |  |  |  |  |  |  |  |  |  |  |  |  |
|  |  |  |  | |  | |  | |  | |  | |  | |  | |
| rs10954214 | TT  TC  CC | 1 123 (44.8%)  1 102 (43.9%)  283 (11.3%) | 69 (46.9%)  60 (40.8%)  18 (12.2%) | | 5.8  5.2  6.0 | | 0.75/0.76/0.61/0.72 | | A 1.01 (0.79-1.28)  A* 0.99 (0.77-1.26) | | 0.97  0.93 | | 1.03 (0.80-1.32)  1.02 (0.79-1.30) | | 0.82  0.89 | |

Considering the risk for the minor allele: A= additive model, D = dominant model, R= recessive model

^a^ HR1=Hazard ratio (95% confidence interval) by Cox proportional hazards survival regression, adjusted for sex, age, BMI, fasting plasma glucose, ACR, smoking status at baseline, and glycemic status at any time. *=same model, plus baseline ACR

^b^ HR2=Hazard ratio (95% confidence interval), same model as HR1 with additional adjustment for systolic and diastolic blood pressures, hypertension, use of diuretics, use of angiotensin-converting enzyme inhibitors or angiotensin receptor blockers, total and HDL cholesterol, triglycerides at baseline. * same model plus baseline ACR
